# Evaluation of Brucellosis Vaccines: A Comprehensive Review

**DOI:** 10.3389/fvets.2022.925773

**Published:** 2022-07-18

**Authors:** Mohsen Heidary, Shirin Dashtbin, Roya Ghanavati, Marzie Mahdizade Ari, Narjess Bostanghadiri, Atieh Darbandi, Tahereh Navidifar, Malihe Talebi

**Affiliations:** ^1^Cellular and Molecular Research Center, Sabzevar University of Medical Sciences, Sabzevar, Iran; ^2^Department of Microbiology, School of Medicine, Iran University of Medical Sciences, Tehran, Iran; ^3^School of Paramedical Sciences, Behbahan Faculty of Medical Sciences, Behbahan, Iran; ^4^Shoushtar Faculty of Medical Sciences, Shoushtar, Iran

**Keywords:** brucellosis, vaccine, *Brucella*, review, *Brucella abortus*, *Brucella melitensis*

## Abstract

Brucellosis is a bacterial zoonosis caused by *Brucella* spp. which can lead to heavy economic losses and severe human diseases. Thus, controlling brucellosis is very important. Due to humans easily gaining brucellosis from animals, animal brucellosis control programs can help the eradication of human brucellosis. There are two popular vaccines against animal brucellosis. Live attenuated *Brucella abortus* strain 19 (S19 vaccine) is the first effective and most extensively used vaccine for the prevention of brucellosis in cattle. Live attenuated *Brucella melitensis* strain Rev.1 (Rev.1 vaccine) is the most effective vaccine against caprine and ovine brucellosis. Although these two vaccines provide good immunity for animals against brucellosis, the expense of persistent serological responses is one of the main problems of both vaccines. The advantages and limitations of *Brucella* vaccines, especially new vaccine candidates, have been less studied. In addition, there is an urgent need for new strategies to control and eradicate this disease. Therefore, this narrative review aims to present an updated overview of the available different types of brucellosis vaccines.

## Introduction

Despite many studies conducted to eradicate brucellosis infection worldwide, the episodic situation of brucellosis is still worrying and ambiguous (1). Brucellosis is a bacterial zoonosis caused by microorganisms belonging to the genus *Brucella*. They are various pathogens of domestic and wild mammals, found inside the host. *Brucella* could multiply in professional and non-professional phagocytes and cause heavy economic losses and many diseases in humans. Controlling brucellosis is of great importance (2). Human brucellosis is caused by direct or indirect contact with various species of infected animals, notably cattle, sheep, goats, and swine. Thus, the wipeout of the illness in animals causes the eradication of human sickness (3). Since the late 1980's, the brucellosis epidemic has been growing rapidly in some countries and parts of the world, infecting over 60 species of wildlife, causing disease worldwide, and causing great economic damage to livestock (4). Humans could easily gain brucellosis through animals and their products, even though humans are not carriers of the disease. Brucellosis is a complex disease due to the diversity of *Brucella* active species that, despite causing species-specific disease syndromes, could sometimes cause cross-infection (5). From the beginning of the twentieth century, the study and research on the production of brucellosis vaccines have begun. The development of brucellosis vaccines has experienced inactivated, live-attenuated, and rough-attenuated vaccines. Inactivated vaccines were first developed to prevent the disease, then live-attenuated vaccines, which are more effective in terms of immunogenicity, were superseded to control brucellosis (6). Existing vaccines that are currently used could cause problems. For example, some of these vaccines could cause human infection and abortion in pregnant cows; however, despite some shortcomings, they play an essential role in preventing and controlling brucellosis. These vaccines are used all over the world. With the development of precise molecular techniques and an accurate understanding of the mechanism of *Brucella* pathogenesis, new genetically-engineered vaccines have been developed and replaced traditional vaccines to prevent and control brucellosis (7, 8). In this review, different types of brucellosis vaccines and their advances evaluated.

## Live-Attenuated Vaccines

In recent decades, the most effective way to control brucellosis has been to vaccinate animals. Although vaccination of individuals living in brucellosis endemic areas, veterinarians, livestock, and laboratory personnel is essential, human vaccines have not yet been developed (9). Live-attenuated vaccines are the most effective vaccines used to control animal brucellosis (10). Due to the lower efficacy of inactivated and subunit brucellosis vaccines, multiple doses should be administered, whilst live-attenuated vaccines are less expensive and more effective and induce immunity through humoral and cell-mediated responses (9, 11). However, some drawbacks have been reported to the administration of live-attenuated brucellosis vaccines, including antibiotic resistance, interference with serological diagnostic tests, and residual virulence in animals and humans (10–12).

Live-attenuated vaccines have been broadly used against brucellosis, such as *B. abortus* strains S19, *B. melitensis* strain Rev1, and M5, and *B. suis* strain S2 derived as an attenuated phenotype by repeated *in vitro* passage of strain 2308. Numerous research on the effectiveness of these vaccines has been carried out in experimental animals and proven that vaccinated animals are effectively protected against wild-type (WT) bacteria. The main disadvantage of vaccine strains S19 and Rev1 is that the agglutinins induced by these vaccines persist in immunized animals for a long time and interfere with the standard serodiagnostic tests, even if the antibodies are produced by these two vaccines are durable. Therefore, it is difficult to distinguish between infected and vaccinated animals with the vaccine strain S19 or Rev1. Although the incidence rate of abortion is low, to overcome these defects, a safe and effective vaccine is needed (13–15). Another vaccine in this category is *Brucella suis* S2 vaccine, which is one of the brucellosis control programs in China. Studies show that this vaccine provides a good humoral and cellular immune response and protects against *Brucella* heterologous species (16), but has a limited host range (17).

Identification of genes linked to virulence or survival of organism's aids to develop new vaccines that are both safe and protective. The best approach to developing new vaccines with minimal residual virulence is currently engineered live-attenuated vaccines based on deletions in virulence genes, which induce high safety levels compared to classical live-attenuated vaccines (18). A variety of vaccines are under development based on different deletions in *B. abortus* or *B. melitensis* virulence genes, which eventually result in significant attenuation and increased production of T cells, pro-inflammatory cytokines, and antibodies. There are many mutants listed in Table 1, which have been generated by attenuation of genes and confer protective responses against *Brucella* challenge in experimental animals.

**Table 1:** Genetically modified live attenuated vaccines against brucellosis.

**Gene deleted**	**Function**	**Host**	**Vaccination (dose, route)**	**Challenge (*Brucella* species)**	**Challenge (weeks or days p.v.)**	**Protection**	**References**
*cydC, cydD, purD*	ATP-binding cassette-type transporter Phosphoribosylamine–glycine ligase	BALB/c mice	2.4–3.1 × 10^8^ CFU, i.p.	*B. abortus* 2308	7w 21w 7w 21w	2.37-log 2.64- log 1.48-log 2.72- log	(19)
*Hfq*	Regular expression of some target genes, affects mRNA stability	BALB/c mice	1 × 10^6^ CFU, i.p.	*B. melitensis* 16M	2w 4w	1.64 -log 2.06- log	(20)
*bp26*	Periplasmic or cytoplasmic protein	BALB/c mice	1 × 10^6^ CFU, i.p.	*B. melitensis* 16M	5w	2.89-log	(21)
*omp31*	Outer membrane protein	BALB/c mice	5 × 10^5^ CFU, i.p.	*B. melitensis* Bm133	3w 6w 9w	≈4.1- log ≈3.9- log ≈2.3- log	(22)
*VjbR*	HTH-type-quorum-sensing-dependent transcriptional regulator	BALB/c mice	1 × 10^6^ CFU, i.p.	*B. melitensis* 16M	2w 4w	1.70-log 3.05-log	(23)
		C57BL/6 mice	10^7^ CFU, i.p.	*B. canis* RM6/66	1 w	3.092- log	(24)
*MucR*	Transcriptional regulatory protein	BALB/c mice	10^5^ and 10^6^ CFU, i.p.	*B. melitensis* 16M	20w	4.14–4.75-log	(25)
*ZnuA*	Zn^2+^ transport system	BALB/c mice	3 × 10^11^ cells, oral	*B. melitensis* 16M	4w	3- log	(26)
*ManB*	Phosphomannomutase (LPS synthesis)	BALB/c mice	1 × 10^6^ CFU, i.p.	*B. melitensis* 16M	2w 4w	1.74- log 1.87- log	(21)
*Pgm*	Phosphoglucomutase (LPS synthesis)	BALB/c mice	1 × 10^6^ CFU, i.p.	*B. melitensis* 16M	2w 4w	3.43-log 2.83-log	(27)

*ip, Intraperitoneal; CFU, colony-forming unit; W, week; log, logarithm*.

Double-deletion (Δ*cydC*Δ*cydD* and Δ*cydC*Δ*purD*) mutants of virulent *B. abortus* induce significant attenuation of virulence and long-term protective immunity. Sera collected from immunized mice with these strains were shown in a study to be associated with significant levels of IgG1 and IgG2a antibodies as well as Th1-type IFN-γ and Th2-type IL-10 cytokines; also, cytokine production was higher in these mice compared to RB51-immunized mice (19). Zhang et al. prepared *B. melitensis* 16M *hfq* (16MΔ*hfq*) mutant strain which induced strong protective immunity, humoral responses especially IgG1 and IgG2a, and cellular responses with IFN-γ and IL-4 cytokine profiles; however, no significant difference in the production of IFN-γ and IL-4 was reported between 16MD*hfq* and Rev1 (20). Another study constructed a *B. melitensis* TcfSR promoter mutant (16MΔTcfSR) to introduce a vaccine candidate against *B. melitensis* infection. TcfSR is one of the two-component regulatory systems which allow host cells to detect environmental variations and respond appropriately to *Brucella*. Induction of a high level of protection and no interference with serodiagnostic tests were the main features of this candidate (28). The M5-90wboA mutant derived from *B. melitensis* M5-90 is a potential attenuated live vaccine and induces less virulence and inflammatory responses compared to its parental strains. The safety of this mutant is evaluated by the lack of splenomegaly in the host. Compared to the original strain, a higher level of protection is provided following vaccination with this mutant (95% survival). Also, another advantage of this mutant is the elicitation of an anti-*Brucella*-specific IgG response following vaccination, which is a diagnostic antigen for differentiation of immunization from infection (10). 16MDwzt as a rough mutant of *B. melitensis*, generated by the disruption of the wzt gene, which encodes the O-polysaccharide (O-PS) export system ATP-binding protein. The level of protection induced by this mutant against *B. melitensis* 16M challenge is similar to that conferred by the *B. melitensis* M5 vaccine. The two advantages of this vaccine are its safety in pregnant animals without inducing abortion as well as its ability to synthesize O-PS without inducing detectable specific antibodies in sheep, which make this vaccine candidate suitable for the eradication of animal brucellosis. The disadvantage reported for this vaccine is its more susceptibility to polymyxin B and complement-mediated killing compared to *B. melitensis* 16M (29). *RM57* is the other Rough attenuated mutant that is generated from *B. melitensis* isolate M1981 has been administered in different animal models (both mice and 186 guinea pigs) and indicated good protective efficacy, especially in guinea pig model. Another advantage of this mutant includes no interference with serological diagnosis. The drawback of this mutant, which could be associated with its reduced virulence in mice and guinea pigs, is its sensitivity to polymyxin B (30). *2308DNodVDNodW* rough vaccine originated from the virulent *B. abortus* 2308 (S2308) by deleting genes encoding a two-component regulatory system (TCS) in chromosome II in S2308. In a study, 2308DNodVDNodW showed significantly reduced survival in murine macrophages (RAW 264.7) and BALB/c mice. In this study, the mutant conferred levels of IgG antibody similar to those conferred by S19; also, a slightly higher level of protection was reported for single- and double-mutant NodVW. This mutant induced a mix of Th1- and Th2-type immune responses as well as strong humoral and cell-mediated immunity in immunized mice. Furthermore, this mutant persisted for a short time in RAW 264.7 macrophages and BALB/c mice. Another advantage of this vaccine is the provision of an ideal diagnostic antigen that could be used to differentiate immunized animals from infected ones (12).

*B. ovis* Δ*abcBA* (*Bo*Δ*abcBA*) vaccine, which has been tested in two formulations (encapsulation with alginate and alginate plus vitelline protein B—VpB), is effective for immunization of mice against *B. melitensis* strain 16M by inducing Th1 (T helper1)-mediated immune responses. Due to its efficacy, the hypothesis of conferring protection against virulent *B. melitensis* in small ruminants could be supported. Also, this vaccine could be administrated for caprine and ovine brucellosis due to *B. melitensis* infection. In rams, this vaccine has an additional advantage, including conferring protection against *B. ovis*, which is another *Brucella* species that commonly infects sheep; immunization with *Bo*Δ*abcBA* against *B. ovis* is highly protective (31). In another study ΔabcBA vaccine could prevent the infection, the secretion of wild-type *B. ovis* in semen and urine of rams, the shedding of neutrophils in semen, and the development of clinical changes and gross lesions induced by wild-type *B. ovis*. This vaccine could induce both humoral and cellular immune responses (32). In a study conducted by Sancho et al., administration of *B. ovis* attenuated mutants (Δomp25d and Δomp22) and *B. melitensis* Rev1 vaccines were compared in mice. The study indicated that mice vaccinated with *B. ovis* mutants developed higher serum levels of anti-*B. ovis* antibodies of IgG1, IgG2a, and IgG2b subclasses as well as IL-1α, as an enhancer of T cell responses to antigen, compared to Rev1-vaccinated mice. Immunization with *B. ovis* mutants indicates appropriate persistence, limited splenomegaly, and protective efficacy against *B. ovis*. Also, *B. ovis* mutants vaccine candidates would likely be the most appropriate vaccines against ram contagious epididymitis (33).

VTRS2 is the other type of rough vaccine which is originated from *B. suis*. This vaccine was constructed by deletion mutations in genes wboA (encoding glycosyltransferase) and leuB (encoding isopropyl malate dehydrogenase). The strain VTRS2 expressing mGnRH can elicit a significant IgG immune response against the mGnRH antigen at 4 and 6 weeks post-inoculation. The rough *B. suis* strain is an effective vaccine candidate in swine (34). *B. suis* Δ *pgm* could stimulate cellular immune responses and induce good levels of protection against the virulent *B. suis* strain, abortion, heifer colonization, and bacterial excretion in milk. Also, using this strain, immunized animals could be differentiated from infected ones. Due to the lack of lipopolysaccharide and the inability to synthesize cyclic beta-glucans, this strain is sensitive to detergents and polymyxin B (35). Compared to the smooth vaccine, the rough mutant strain of *B. neotomae* stimulates further activation of dendritic cells *in vitro* and confers protection against the heterologous challenge by *B. suis* in mice (36).

*B. abortus* 2308 ery promoter mutant (Δery) safety is evaluated by the lack of splenomegaly in inoculated mice. This vaccine has good protective efficacy and could induce the secretion of higher levels of IFN-γ and IL-4 compared to S19. Post-vaccination humoral responses provide an ideal diagnostic EryA antigen for the differentiation of immunization from infection using EryA-iELISA. Also, sensitivity to erythritol and reduced survival in macrophages and BALB/c mice could be observed in this vaccine (37). *IVK15*Δ*cydD and IVK15*Δ*cydC* mutants are created by deleting only *cydD* and *cydC* genes, encoding ATP-binding cassette transporter proteins, from the chromosome of the virulent *B. abortus* strain isolated from Korean cow (referred to as IVK15). Mice immunization with these mutants could protect them against the virulent *B. abortus* strains and S2308. Also, higher levels of anti-*Brucella*-specific IgG, IgG1, and IgG2a antibodies and higher levels of IgG2a than IgG1 could be observed in immunized mice compared to unvaccinated mice. Splenomegaly is a consequence of inflammatory responses is not observed in immunized mice with IVK15Δ*cydD* and IVK15Δ*cydC*. Both mutants exhibit increased sensitivity to metal ions, acidic pH, and hydrogen peroxide, which resemble the intracellular environment during host infection (11). The *B. abortus* S2308 mutant strain Δ22915 is constructed by deleting the putative lytic transglycosylase gene BAB_RS22915. This mutant induces an effective immune response with fewer inflammatory responses. Higher levels of antibody and better protection against *B. abortus* S2308 are induced by Δ22915 mutant compared with RB51 (12). Several mutants listed in Table 2, such as Δ*mucR* and Δ*vjbR*, have been studied to evaluate the level of protection and the ability to induce humoral and cellular responses (23–25). Understanding the immune responses and protective mechanisms against *Brucella* infection is important for the development of an effective vaccine. T-cell subsets and antibody responses are necessary to confer protection against virulent stains. Cytokine profiles, including TNF-α, IFN-γ, IL-1, and IL-12, contribute to controlling *Brucella* infection in its early stages. Therefore, inducing a high level of immune system responses contributes to the effectiveness of vaccines and should be considered in vaccine development (14). The main features of these vaccine candidates are mentioned in Table 2. However, in all the reviewed studies, the positive aspects of these candidates have been mentioned, but the drawbacks of these candidates must also be considered, including not complete elimination of persistent strains (44) or the risk of spreading antibiotic resistance in cloning procedures. In addition, they should be evaluated in livestock and trial studies (45).

**Table 2:** Introduced genetically engineered live-attenuated vaccines.

**Name of vaccine**	**Advantage**	**References**
Double deletion mutants of *B. abortus* (BA15Δ*cydC*Δ*cydD* and BA15Δ*cydC*Δ*purD*)	- Incapability of intracellular survival and replication within macrophages - Attenuated virulence and limited persistence in the host - Conferring long-term protection in mice - Inducing significant levels of IgG antibodies - Inducing significant amounts of IFN-γ and IL-10 - Conferring a high level of protection with each mutant - Level of safety	(19)
*B. melitensis* 16MΔ*hfq*	- Attenuated virulence and limited persistence in the host - Inducing significant levels of IgG1and IgG2a antibodies - Inducing higher amounts of IFN-γ and IL-4 - Level of safety - Conferring a high level of protection	(20)
*B. melitensis* 16MΔTcfSR	- Conferring a high level of protection - Significantly inducing higher IgG levels - Inducing higher amounts of IFN-γ - Inducing high levels of IgG - Differentiation between the vaccination and infection	(28)
*B. melitensis* M5-90Δ*bp26*	- Conferring slightly better protection than M5-90 - Low virulence and higher immunoprotectivity following 16M strain challenge - Inducing higher amounts of IL-6 and TNF-α - Eliciting an anti-*Brucella*-specific IgG response	(21)
*B. melitensi*s LVM31 mutant strain	- Conferring protection similar to that induced by the *B. melitensis* Rev1 vaccine strain - Decreasing splenic colonization - Presenting no lesions or apparent histopathological changes - Significantly lower persistence of bacteria in the spleen	(22)
*B. abortus* IVKB9007 *looP*::Tn*5* and *cydC*::Tn*5*	- Conferring a high level of protection - Significantly attenuated virulence	(38)
*B. melitensis* 16MΔ*hfq*	- Conferring a high level of protection - Inducing higher amounts of IFN-γ and IL-4 - Downregulating the expression of IL-2 and IL-10 in mice in the 16MΔhfq group, while upregulating expression of IL-4 and IFN-γ - Significantly inducing higher antibody levels in the *hfq* mutant-immunized mice at 14 and 28 days post-challenge compared to the PBS group as control	(39)
*B. melitensis* M5-90Δ*vjbR*	- Reduced survival capability in macrophages - Conferring a high level of protection - Serological differentiation between infected and vaccinated animals - Significantly attenuated virulence - Inducing significant levels of IgG antibodies - Significantly inducing higher amounts of IFN-γ and IL-4	(23)
*B. canis* RM6/66 Δ*vjbR*	- Conferring a significant level of protection against organ colonization and development of histopathologic lesions following intraperitoneal challenge - Inducing a significant increase in IgG1 and IgG2a levels - Significantly inducing higher levels of IFN-γ	(24)
*B. melitensis* 16MΔ*mucR*	- Conferring a significant level of protection following both intraperitoneal and aerosol challenge - Absence of *Brucella* associated pathological changes, including splenomegaly, hepatomegaly, or granulomatous disease - Eliciting a strong protective immunity - Significantly reducing the colonization compared to the parental strain	(25)
*B. melitensis* Δ*znuA*	- Oral live vaccine candidate Δ*znuA B. melitensis* induces protection against nasal challenge with wt *B. melitensis* 16M - Rapid clearance from mice within 2 weeks - Conferring an effective protection in mice upon nasal challenge - Enhancing clearance of *Brucella* from the lungs and spleen - Inducing both systemic and mucosal Th1 and Th17 responses, while Th17 produces IL-17 and IL-22	(26)
*B. melitensis* M5-90Δ*manB*	- Significantly reduced survival in macrophages and mice - Inducing a strong protective immunity in BALB/c mice - Eliciting anti-*Brucella*-specific IgG1 and IgG2a subtype responses - Inducing the secretion of IFN-γ and IL-4 - Serological differentiation between infected and vaccinated animals	(21)
*B. melitensis M5-90Dpgm*	- Significantly reduced survival in embryonic trophoblast cells and in mice - Conferring a high protective immunity in BALB/c mice - Eliciting an anti-*Brucella*-specific immunoglobulin G response - Inducing the secretion of IFN-γ and IL-2 - Serological differentiation between infected and vaccinated animals - Inducing the secretion of IFN-γ in immunized sheep	(27)
*B. abortus* Δ*norD* Δ*znuA*	- Highly attenuated in mouse and human macrophages - Complete clearance from mouse spleens within 8 weeks post-vaccination - Significantly inducing more protection than the conventional RB51 vaccine - Significantly inducing higher levels of IFN-γ and TNF-α - Conferring a high level of protection	(40)
*B. ovis* ΔabcBA	- Preventing the infection, the secretion of wild type *B. ovis* in semen and urine, the shedding of neutrophils in semen - Development of clinical changes as well as gross and microscopic lesions induced by wild type *B. ovis* reference strain - Inducing humoral and cellular immune responses	(32)
*B. ovis* (Δomp25d and Δomp22)	- *B. ovis* mutants: appropriate persistence, limited splenomegaly, and protective efficacy against *B. ovis*- *B. ovis* attenuated strain: probably the most interesting candidate to develop a specific vaccine against ram contagious epididymitis - *B. ovis* mutants: developing anti-*B. ovis* antibodies in serum - *B. ovis* Δomp25d: representing a spleen colonization profile similar to that of *B. melitensis* Rev 1 and *B. ovis* Δomp22 and eliciting only a moderate degree of splenomegaly - *B. ovis* Δomp25d and Δomp22: inducing protective activity and a limited degree of splenomegaly	(33)
*B. ovis* IVK15Δ*cydD* and IVK15Δ*cydC*	- Reduced intracellular survival in macrophages - Wild-type IVK15 induces splenomegaly due to inflammatory responses, but not IVK15Δ*cydD* and IVK15Δ*cydC*. - Rapid elimination from the spleens - Significantly inducing higher levels of *Brucella*-specific IgG, IgG1, and IgG2a responses mostly induced by Th1 - Possessing sufficient immunogenic properties to confer protective immunity in mice against *B. abortus* infection - Markedly attenuated virulence both *in vitro* and *in vivo*	(11)
*B. abortus* mutant strain Δ22915	- Inducing fewer inflammatory responses than the wild-type strain - Inducing an effective immune response against the wild-type strain S2308. - Decreasing bacterial loads after vaccination for up to 4 wpv - Increasing specific antibody titers to a peak at 12 wpv - Inducing higher levels of antibody and providing longer and better protection against *B. abortus* S2308 than RB51 - Significantly attenuated virulence of the mutant strain Δ22915	(12)
*B. melitensis* M5-90 wboA	• Faster response • Safety • Reduced virulence and inflammatory response • Inducing a high level of protection • Suitable live vaccine candidate	(10)
*B. abortus* 2308DNodVDNodW	• Significantly reduced virulence • Inducing a slightly higher level of protection than the *B. abortus* vaccine S19 • Inducing a mix of Th1- Th2, humoral, and cellular immunity • Persistence for a short period of time in RAW 264.7 macrophages and BALB/c mice, thereby reducing virulence of *Brucella* • Suitable live vaccine candidates	(41)
*B. canis vjbR* mutant strain	• Inducing no impairment in bacterial growth rate or obvious pathological damage • Inducing a considerable protective immune response against *B. canis* RM6/66 strain	(42)
*B. abortus* 2308ΔgntR	• Inducing humoral immunity, cytokine responses, and high protective immunity against the virulent strain • Eliciting an anti-*Brucella*-specific immunoglobulin G (IgG) response • Inducing the secretion of IFN-γ and IL-4	(43)
*B. suis* Delta-pgm	• *B. suis* pgm strain is able to trigger a robust cellular immune response. • Inducing a significant level of protection against the virulent *B. suis* • Inducing high levels of protection against abortion, heifer colonization, and excretion in milk • Replication in cultured cells • Completely avirulent in the mouse model of infection, but inducing protection against the virulent strain challenge • Inducing the production of pro-inflammatory cytokines	(35)

*IFN, Interferon; TNF, Tumor necrosis factor; IL, interleukin; Th, T helper; IgG, Immunoglobulin G*.

## Vector Vaccines

Recently, various viral or bacterial vector-based *Brucella* vaccines have been fostered as efficient delivery systems to deliver different heterologous or homologous antigens (46). They are live vector-based genetically modified vaccines (47). Cell-mediated immune responses induced by intracellular organisms may represent that the best choice is to present *Brucella* antigens to the immune system of the target host; the main goal of these candidates is to promote the formation of an antigen-specific T-cell immune response (48). These types of vaccines replicate in the host cell, producing multiple copies of the *Brucella* antigen (49). There are various bacterial or viral vectors for the expression of *Brucella* proteins, including Lactococcus *Lactis, Escherichia coli, Salmonella* strains, or influenza virus (47, 50). Each of these vectors has several advantages and disadvantages. *Salmonella*, as an intracellular pathogen, delivers antigens effectively to antigen-presenting cells such as macrophages. Other advantages of using *Salmonella* as a vector include inherent adjuvant effect, adequacy of a single-dose vaccination to obtain long-lasting immunity, the ability to multiply and present multiple antigens, and dynamic entrance into the natural barrier protecting antigens from host degenerative enzymes. Some research studies have indicated that multiple infections caused by *Salmonella* could lead to increased disease outcomes in infected animals. The potentiation of this pathogenesis may be due to the immunomodulatory effect of *Salmonella*, which inhibits or delays the host immune response and promotes systemic *Salmonella* infection. In acute conditions, salmonellosis could also cause miscarriage and death, which could lead to reduced animal productivity (51). Influenza viral vectors (IVV) have also been developed due to the lack of pre-existing immunity against H5N1 influenza virus in the human population (47). There is a confirmed IVV-based *B. abortus* vaccine (Flu-BA) developed in Kazakhstan for cattle vaccination; although bovines are not highly susceptible hosts to influenza A virus infection, and there is a natural immunity to influenza infection in this host. However, it could be more effective for humans because influenza A is a common human infection. There is widespread concern about the use of IVV of the H5N1 subtype, which is a pathogenic influenza virus spreading in poultry. The main concern is related to the interspecies transmission of the disease from birds to humans, which could lead to human disease. Although the replication capacity of this virus has been limited in this vaccine by eliminating the proteolytic cleavage site in HA, the risk of pandemic strains must be considered (52). Lactic acid bacteria (LAB) are also considered a desirable antigen delivery system for mucosal immunization. It has been reported that *L. casei*-based vaccines show a protective response against challenges (53). Recently, mucosal vaccination has been considered because the main route of natural transmission of brucellosis is usually through mucosal exposure. One of the disadvantages of using live LAB-based mucosal vaccines is related to the risk of spreading genetically engineered organisms carrying drug resistance markers to the environment and the host flora. In addition, *L. lactis* strains are considered to be non-colonizing bacteria that survive when passing through the gastrointestinal tract (GIT) and trigger immune responses when taken up by M cells (54). Adenovirus-based vaccines are another type of vector vaccine with several disadvantages including high levels of pre-existing immunity, transient expression of the transgene, and highly immunogenic (55). Moreover, due to the complexity of the target pathogen, multiple antigens are required to enhance effective immune responses, which incur more clinical evaluations and higher manufacturing costs (56).

Different antigens are used for developing this type of vaccine, such as proline racemase subunit A (PrpA), Cu/Zn superoxide dismutase (SOD), *Brucella* lumazine synthase (BLS), lipoprotein outer-membrane protein 19(Omp19) (57), and ribosomal protein L7/L12 (58). These antigens efficiently induce immune responses restricting the pathogen in the early stages of infection. The function of antigen-presenting cells such as macrophages and dendritic cells is to stimulate the production of specific antibodies, T cells responses such as CD4^+^ and CD8^+^, and the secretion of cytokines involved in bacterial resistance and elimination. BLS, Omp19, PrpA, and SOD could efficiently induce the secretion of Th1-type cytokines. PrpA also stimulates B cell responses. Omp19 induces Th1 responses and mouse dendritic cell maturation. In a study, an attenuated *S. typhimurium* strain expressing BLS, Omp19, PrpA, or SOD of *B. abortus* in goats was shown to elicit strong cell-mediated immune responses against PrpA, BLS, Omp19, and SOD, but greater humoral responses were elicited against Omp19 and SOD. This type of vaccine could provide a high level of protection for individual groups. Regardless of high protection, this type of vaccine requires multiple boosters and adjuvants to obtain long-lasting immunity, but without affecting bacterial viability (57). *Brucella* ribosomal protein L7/L12 has a high antigenicity due to the dominant epitopes. The combination of protein L7/L12 with *Salmonella* delivery system (JOL1800 strain) induces humoral and cell-mediated immune responses. High numbers of stimulated cells, including CD4^+^ and CD8^+^ T cells, and the production of IFN-γ have been reported in L7/L12-immunized mice. Besides the high antigenicity of L7/L12, the JOL1800 strain has a high level of safety, and a single dose of vaccine effectively eliminates the pathogen (58). Oral administration of attenuated *Salmonella* strain secreting *Brucella* antigens, including Cu–Zn superoxide dismutase (SodC) and outer membrane protein 19 (Omp19), with sodium bicarbonate antacid, significantly induces the secretion of a high level of systemic IgG and a mixed Th1–Th2 response. The rate of *Salmonella* colonization following the development of this type of vaccine has increased, stimulating protective immune responses (59). Attenuated *Salmonella* strains expressing *B. abortus* BCSP31, Omp3b, and superoxide dismutase proteins have also been investigated as a vaccine candidates (60).

Numerous recombinant viral vector vaccines have been evaluated so far. In a study, an influenza viral vector of the H5N1 subtype, as a non-replicable viral vector, expressing *Brucella* Omp16, L7/L12, Omp19, and Cu–Zn SOD immunodominant proteins was investigated in guinea pigs against human brucellosis. Although no immune response was reported in this study, different administration routes and vaccine doses were evaluated. To determine the best immunization route, different routes were evaluated, such as conjunctival (c.), intranasal (i.n.), and sublingual (s.l.). A significant protective effect was reported for this vaccine when administered through i.n. (2.8 log _10_) and c. (2.3 log _10_) administration routes, comparable to *B. melitensis* Rev1 vaccine results; also, the optimum dose conferring a high level of protection was determined to be 10^6^ EID50 and 10^7^ EID50 (47). Recently, Bugybayeva et al. suggested the tetravalent vaccine formulation Flu-NS1-80-Omp16+Flu-NS1-80-L7/L12+Flu-NS1-80-Omp19+Flu-NS1-80-SOD to develop a safe and effective human vaccine. In this study, a recombinant influenza viral vector (rIVV) of H5N1 subtype, expressing *Brucella* L7 / L12, Omp16, Omp19, or Cu-Zn SOD immunodominant protein containing a sequence of 80 N-terminal amino acids from the open reading frame (ORF) of the NS1 gene, was evaluated. The results of this study indicated that this formulation had a high level of safety and efficacy. This research is an important report on the development of a safe and protective vaccine against human brucellosis (52). In another study, recombinant influenza A viruses of the subtypes H5N1 and H1N1, expressing L7/L12 or Omp16, were developed and shown to elicit Th1 CD4^+^ and CD8^+^ T-cell immune responses and confer good protection against challenge (61). The expression of BP26 as a highly conserved immunogenic protein in *Brucella* by pseudorabies virus was also screened as a vaccine candidate in another study by Yao et al. This type of vaccine can induce humoral and cellular immune responses. The extensive tropism of this vaccine makes it a suitable vector (62). Guo-Zhen et al. reported that Adenovirus-LL/BP vaccinated mice had higher levels of IgG, IgG1, and IgG2a antibodies. Their study results indicated that this vaccine-induced primarily cellular and partially humoral immunity and provided a mild protection level against *B. abortus* infection. Although this type of vaccine conferred significant protection against challenge, the level of protection was lower compared to the live A19 vaccine (55). As mentioned earlier, probiotics such as *L. casei* are considered as a vector to elicit a good immune response and a high level of protection comparable to that induced by the IRIBA Strain Vac Calf vaccine. *L. casei* strains expressing the outer membrane protein OMP19 prompt Th1/Th2 immune responses and the production of IFN-γ, IL-2, and IL-4. As *Brucella* is an intracellular pathogen, cell-mediated immune responses are required to control the pathogen. Therefore, immunodominant antigens should be considered in developing new vaccines to stimulate cellular immune responses. In this regard, the production of cytokines such as IFN-γ, IL-2, and IL-4 is critical (53). The initial step of infection occurs in mucosal areas; thus, mucosal vaccination could be done to elicit a good response. In this context, mucosal administration of *L. casei* or *L. lactis* vector vaccines, generally regarded as safe, is a potential vaccine delivery approach. It has been suggested that the danger of eliciting immunological tolerance may also be faded compared with the persistent strains (63). Although other viral and bacterial vectors have been investigated, it should be noted that to introduce a safe vaccine, the non-pathogenicity of organisms must be proven (Table 3).

**Table 3:** Characteristics of the vector-based vaccines.

**Vector**	**Antigens**	**Host**	**Challenge**	**Advantage**	**Disadvantage**	**References**
*S. typhimurium*	BLS, Omp19, PrpA, or SOD	Goat	*Brucella* strain- HJL254	1. Safety of vaccine 2. Higher titers of IgG against Omp19 3. Successful delivery of Omp19 4. Higher production of IFN-γ in SOD stimulated goats 5. A significant level of protection with individual antigens in vaccine 6. A strong cell-mediated immune response	1. Low levels of anti-PrpA and -BLS IgG 2. Limited scope of efficacy of this vaccine (generally < 2 log10 units) 3. Several boosters would be required to achieve a long-term immunity.	(57)
*S. typhimurium* JOL1800	Ribosomal protein L7/L12	Mice	*B. abortus* 544	1. Efficient elicitation of both IgG (IgG1 and IgG2a) and sIgA 2. A significant increase in IFN-γ and IL-4 expression levels 3. A significant increase in both CD4^+^ and CD8^+^ expressing cells 4. Enhancing the chance of antigen presentation by *Salmonella* secreting L7/L12 antigen 5. Clearly inducing both IgG and IgA by a single dose 6. JOL1800 strain induces no mortality in immunized mice due to attenuation by deletion of *lon, cpxR*, and *rfaL* genes. 7. Minimum pre-existing lipopolysaccharides (LPS)-specific anti-*Salmonella* immunity in the host	NR	(58)
*S. typhimurium* (ST) strain JOL1800	Cu–Zn superoxide dismutase (SodC) and outer membrane protein 19 (Omp19)	Mice	*B. abortus* strain 544	1. Enhancement of humoral and cellular immune responses and subsequent protection due to the use of sodium bicarbonate antacid formulation 2. PH buffering action around the neutral values could be particularly an advantage for the present vaccine strain to produce an effective immune response. 3. Increasing the number of *Salmonella* in the intestinal environment 4. Activation of both Th1 and Th2 antibody responses	NR	(59)
HJL228, HJL219, and HJL213	BSCP31, Omp3b and superoxide dismutase	Mice	*B. abortus* strain 544	1. Significantly inducing higher serum levels of IgG, TNFα, and IFN-γ in group E (immunized with ~1×10^6^ CFU) 2. Significantly inducing higher levels of TNF-α in response to all antigens in groups D (immunized with ~1×10^5^ CFU) and E 3. Significantly inducing higher levels of IFN-γ in response to all antigens in groups D and E than in groups A (immunized with PBS) and B (immunized with *Salmonella* containing vector only)	NR	(60)
Influenza viral vectors (rIVV) subtypes H5N1	Omp 16, L7/L12, Omp19, or Cu–Zn SOD	Guinea pigs	*B. melitensis* 16 M	1. Inducing a significant protection after intranasal (*i.n*.) administration of the vaccine 2. Comparability of the protection level induced by conjunctival (*c*.) administration route to that induced by the commercial *B. melitensis* Rev1 vaccine 3. Inducing the highest level of protection (vaccination efficiency) against the infection in guinea pigs immunized at doses of 10^6^ EID50 and 10^7^ EID50 (80%) compared with the control group (PBS) after the challenge	NR	(47)
Influenza viral vector (rIVV) subtype H5N1	Omp 16 and 19, ribosomal L7/L12, and Cu-Zn superoxide dismutase (SOD)	Mice and guinea pigs	*B. melitensis* 16M	1. Tetravalent formulation is a safe vector, and its protective efficacy against *B. melitensis* 16M infection in the prime-boost regimen is comparable to that induced by the commercial *B. melitensis* Rev1 vaccine in mouse and guinea pig models.	NR	(52)
Influenza viral vectors (IVV) subtypes A/H5N1	Omp16, L7/L12, Omp19, or Cu-Zn superoxide dismutase (SOD)	Sheep and goats	*B. melitensis* 16M		NR	(50)
Pseudorabies virus	BP26	Mice	NR	1. The virus is infective and fatal for most livestock. 2. Its multiple species tropism makes PRV vaccine virus as one of the best vectors to develop bivalent or trivalent vaccines.	NR	(62)
Adenovirus	L7/L12 and BCSP31	Mice	*B. abortus* strain CVCC12	1. Eliciting higher IgG, IgG1, and IgG2a antibody levels 2. Inducing high levels of IL-12 (Th1-type cytokine) and IL-10 (Th2 type cytokine)	Weaker efficacy of this vaccine than that of the live A19 vaccine	(55)
*L. casei*	OMP19	Mice	*B. abortus* 544	1. Increasing serum levels of IFNγ, IL-2, and IL-4 2. Immunization with recombinant *L. casei*- OMP19 prompts a mixed Th1/Th2 immune response. 3. Significantly inducing a high level of protection 4. Comparability of the protection level obtained with recombinant *L. casei* to that acquired by the IRIBA Strain Vac Calf vaccine	NR	(53)
*L lactis*	Cu,Zn superoxide dismutase	Mice	*B. abortus* 2308	1. Inducing protective immune responses at the mucosal level 2. Eliciting agent-specific immunity at the systemic level 3. Induction of systemic and mucosal SOD specific-immune responses in mice orally immunized with *L. lactis* genetically modified to secrete SOD	NR	(63)

*NR, not reported; IFN, Interferon; TNF, Tumor necrosis factor; IL, interleukin; Th, T helper; IgG, Immunoglobulin G; Omp, outer membrane protein; SOD, Superoxide dismutase; sIgA, Secretory Immunoglobulin A; CFU, colony-forming unit; PRV, Pseudorabies virus*.

## Subunit Vaccines

Brucellosis is a chronic zoonotic disease that is mainly transmitted from animals to humans and could pose significant risks to public health and safety. *Brucella* spp. but only is an intracellular pathogen that survives within neutrophil leukocytes without inducing significant activation, also strongly resistant to the bactericidal action of antimicrobial peptides and serum (64). Thus, the successful development of brucellosis vaccines is a major challenge. Vaccination is a major policy decision to prevent both animal and human brucellosis. The subunit vaccines are promising vaccine candidates due to their safety profile, well-defined non-infectious nature, inability to revert to a virulent strain, non-viability unlike attenuated vaccines, ability to induce the production of high levels of antibody, and capability of manipulation to maximize desirable activities. The formulation of these vaccines is the use of a recombinant highly-conserved protein that could affect multiple *Brucella* species. However, they could not replicate and mimic a natural *Brucella* infection (tissue and cell tropism) and therefore provide a lower protective efficacy compared with live-attenuated vaccines (65). The poor antigenicity, instability, and short half-life of recombinant subunit antigens are the main impediments in the design of an effective subunit vaccine against brucellosis (66). In this context, the use of adjuvants, immunomodulators, antigen delivery systems, or TLR (toll-like receptor) ligands is necessary to enhance well-balanced immune responses. The type of induced immune response depends on the type of antigen and adjuvant used in recombinant *Brucella* protein vaccines. Freund's adjuvant (the most commonly used adjuvant), Alum adjuvant, and aluminum hydroxide (the only adjuvant licensed for use in human vaccines) generate Th2-type immune responses, while monophosphoryl lipid A and CpG induce Th1-type responses. To screen and evaluate protective antigens, a combination of an appropriate antigen, adjuvant, booster, and delivery vehicle/vector is needed to trigger a strong protective immune response, such as the Th1 immune response as the dominant immunity against brucellosis (44). For the development of an effective vaccine against intracellular pathogen represented by *Brucella*, the production of Th1- derived cytokines (IL-12, TNFα, IFNγ) as well as the activation of macrophages, dendritic cells, and CD4+ and CD8+ T cells are the key factors for the clearance of infection; whereas Th2 immune responses, which are induced by the humoral immune system, have a minor role in the clearance of infection (67). Cytokines play a main role in the development, maturation, differentiation, and activation of immune cells. For instance, IL-4 (Th2 cytokines) induces IgG1 antibody formation by differentiation of naive CD4+ T cells into Th2 cells, whereas IFN-γ (Th1 cytokines) induces IgG2 antibody formation by differentiation of naive CD4^+^ T cells into Th1 cells (68). IL-10 is an immune-regulatory cytokine that induces the balance of Th1 or Th2 immune responses to prevent over activity of the immune system and limit further tissue damage (69). Numerous cell surface and intracellular components could be expressed by *E. coli* and serve as protective antigens in mouse models, such as outer Omp2b, OMP16, OMP19, L7/L12 ribosomal protein (70–72), Omp31 (73), outer membrane protein Omp25 (71), p39 (a putative periplasmic binding protein) (74, 75), AsnC (76), Omp16 (77), lumazine synthase (78), rE2o (79), rCysK (80), DnaK (81), chimeric protein from OMP19 and p39 domains (75), OMP25-BLS fusion protein (82), OMP25c protein mixed with freund's adjuvant (83), and AspC, Dps, lnpB, and Ndk (84); however, none of them have shown a successful clearance. Previous studies have shown that combining several recombinant proteins which generate a wide array of immunogenicity could induce stronger immune responses and better protection against *Brucella* than their univalent counterparts (74, 85, 86). Also, several studies have shown that subunit vaccines could induce protection levels and immune responses similar to those induced by live or attenuated vaccine strains (69, 72, 73, 83, 84, 87–89). At the same time, other studies have not observed such findings (90). There is a wide range of factors influencing immune responses and protection induced by vaccination in the mouse model, including intrinsic host factors (sex, age, and type of mice), vaccine factors (such as vaccine type, adjuvant type, number and dose of vaccination), administration factors (schedule, site, route, time of vaccination), and challenge factors (challenge pathogen strain, route, challenge-killing interval, time interval between vaccination and challenge and/or between challenge and assessment of splenic bacterial loads) (91, 92). Although subunit vaccines have the advantage of safety, they require multiple boosters and a combination of several antigens, adjuvant, and delivery vehicle/vector to induce an effective immunity and protection against brucellosis in cattle, which isn't economically viable (44). Moreover, it is important to consider those immune responses elicited in mice may not accurately reflect the protection and immune responses elicited in natural hosts after vaccination. Therefore, more extensive studies are needed to identify new recombinant vaccines containing more than one *Brucella* antigen. Unfortunately, no successful subunit vaccine for brucellosis has been developed so far despite many efforts (Table 4).

**Table 4:** Subunit vaccine regimens and protective efficacies.

**Type of vaccine**	**Name of vaccine**	**Properties**	**Immunization dose/ route**	**Comparator/** **route**	**Challenge stain/ dose/ rout**	**Adjuvant**	**Booster**	**Interval**	**Humoral immune response**	**Cellular immune response**	**Lymphocyte bias**	**References**
Gene code (vector)	1. rBP26 2. rOmp25 3. rL7/L12 4. rBP26 + rOmp25 + rL7/L12	26 kDa periplasmic protein, 25 kD OMP, ribosomal protein	40 μg, 30 μg, 40 μg/i.p	S19/i.p	*B. abortus* 544 / 2 × 10^5^/-	Alum	Yes	2 wks	IgG1↑ IgG2a↑	TNF-α ↑ IFN-γ ↑ IL-10 ↑	Mixed Th1/Th2	(71)
Multi-epitope protein (B cell epitopes and T cell epitope) bioenf	rMEP	Epitope of rOMP16 rOMP2b rOMP31 BP26	30 μg	*B. melitensis* M5-90 /1 × 10^9^/s.c	*B. melitensis* 16 M / 5 × 10^5^/i.p	CFA/IFA	-	2 wks	IgM↑ IgG1↑ IgG2a↑ IgG2b↑ IgG3↑	IFN-γ ↑ IL-6 ↑	Mixed Th1/Th2	(77)
Gene code	1. rTF+ 2. rBp26+ 3. rOmp31	Trigger factor, 26 kDa periplasmic protein, 31 kD OMP	20 μg, 30 μg, 40 μg/s.c	RB51/ 2×10^8^/i.p Rev1/ 2×10^8^/i.p	*B. abortus* 544/ 2×10^5^/i.p *B. melitensis* 16M/ 2×10^5^/i.p	CFA/IFA	-	2 wks	IgG1↑ IgG2a↑	IFN-γ ↑ IL-4↑ IL-10↑ IL-12↑	Th1	(73)
Recombinant proteins	1. rPGM 2. rDapB	Enzyme	30 μg	-	*B. abortus* (S2308) (invitro)	CFA/IFA	-	2 wks	IgG1↑ IgG2a↑	IFN-γ ↑ IL-2 ↑ IL-4 ↑ IL-5 ↑	Th1	(93)
Protein	OMV	OMV *B. abortus S* 99	5 μg/sc	S19 / 1× 10^4^/ i.p	*-*	CFA/IFA	Yes	2 wks	-	-	-	(94)
Recombinant proteins	1- Omp10-Omp28-L7/L12 (*P. pastoris* /*E. coli*) 2- Omp10-Omp28-L7/L12+ adjuvant (*P. pastoris* /*E. coli*) r Omp10 3- rOmp28 4- rL7/L12	Lipoprotein, soluble protein or BP26, ribosomal protein	0.1 mg of each/	*B. melitensis* M5 /5 × 10^4^/i.p	*B. melitensis* 16M/ 5 × 10^5^/i.p	TPPPS	Yes	1 wk	IgG1↑ IgG2a↑	IFN-γ ↑ IL-2 ↑ IL-4 ↑	Th1	(70)
DNA vaccine	1. rTOmp2bpcDNA3.1 2. TOmp2bpcDNA3.1 3. TOmp2b priming/ rTOmp2b boosting	Truncated 36 kDa Omp	rProtein: 30 μg/s.c plasmid: 50 μg/s.c	Rev1/ 2 × 10^8^/ i.p, RB51/ 2 × 10^8^/ i.p	*B. abortus* 544/ 4×10^4^/ i.p, *B. melitensis* 16 M/ 2× 10^4^/ i.p/	Montanide ISA 70VG + CpG ODN 1826	Yes	3 wks	IgG1↑ IgG2a↑	↑ IFN-γ ↓ IL-10 IL-4↓	Th1	(95)
Recombinant proteins	1. rL7/L12-rTOmp31-rSOmp2b+ Poly (I:C) 2. rL7/L12-rTOmp31 -rSOmp2b+ CpG+ Montanide	Ribosomal protein Truncated 31 kDa Omp Truncated 36 kDa Omp without the signal peptide	rProtein: 20 μg of each/s.c Adjuvant: 50 μg	Rev1/ 2 × 10^8^/i.p RB51/ 2 × 10^8^/i.p/	*B. abortus* S 544/ 4 × 10^4^/i.p *B. melitensis* S16 M/ 2 × 10^4^/i.p	CpG ODN 1826+ Montanide ISA 70VG / Poly (I:C)	-	3wks	IgG1↑ IgG2a↑	IFN-γ ↑ IL-2 ↑	Th1	(72)
Gene cod	rBCG-P39-L7/L12	39-kD periplasmic binding protein, Ribosomal protein	4 × 10^8^ CFUs/s.c	PBS	*B. melitensis* M28/ 5× 10^5^/i.p	-	-		IgG1↑ IgG2a↑	IFN-γ ↑ IL-4↑ IL12p70↑ TNF↑	Th1	(96)
Recombinant proteins	1. rOMP25 2. rHSP60 3. rOMP25+BLS 4. rOMP25-BLS+ hsP60	25 kD OMP, enzyme, heat shock protein 60 kDa	20 μg, 40 μg, 30 μg/i.p	Rev1 / 1–4× 10^9^/i.p	-	IFA AH CS-NPs	Yes	2 wks	IgG1↑ IgG2a↑	IFN-γ ↑ TNF-α↑ IL-4↑	Th1	(82)
Protein	S19-OMP-liposome	OMP of *B. abortus* strain S19, vaccine delivery system	50 μg/s.c	S19/1.1 × 10^5^	*B. abortus* 544/ 2.2 × 10^5^/i.p	-	-		IgG1↑ IgG2a↑	-	Th1	(97)
Multi-epitope subunit (gene code)	1- FliC +7α-HSDH + BhuA 2- FliC + 7α-HSDH + BhuA without Adjuant	Flagellin Enzyme Heme transporter	Poly B= B cell and T CD4+ epitopes/ Poly T=T CD8+ and T CD4+ cell epitopes Dose (N.D)	RB51 Rev1	*B. melitensis* 16M *B. abortus* 544/ 2×10^7^/ i.p	Poly I:C	-	2 wks	IgG1↑ IgG2a↑	IFN-γ ↑ IL-2↑ IL-10	Th1	(87)
Protein	1- OMVs only 2- OMVs + Poly(I:C) 3- OMVs + CpG ODN + Montanide ISA 70VG	OMV of B. *melitensis strain* 16 M	5 μg/s.c	Rev1/ 2 × 10^8^/ i.p	*B. melitensis* / 2 × 10^4^/i.p	Poly(I:C)/CpG ODN 1826/Montanide ISA 70VG	-	3 wks	IgG2a ↑ IgG1↑	IFN-γ ↑ IL-2↑	Th1	(88)
DNA vaccine	1- pCIOmp31 2- pCIOmp31 + adjuvant	31 kDa Omp Enzyme	100 μg/i.m	HKBC *B. canis* /1 × 10^9^/s.c	*B. canis* RM6/66/ 5.5 × 10^5^/i.p	IFA AH Montanide Quil A	Yes	2 wks	IgG↑	IFN-γ IL-4	Mixed Th1–Th2	(98)
Gene cod (recombinant proteins)	RHspA	Heat shock proteins	30 μg/i.p	Rev1 /8 × 10^8^/ s.c	*B. melitensis* 16 M/ 1 × 10^4^/i.p	CFA/IFA	Yes	2 wks	IgG1↑ IgG2a↑	IFN-γ↑ IL-12↑ IL-6 ↑ IL-10↑ IL-4↑ IL-5↑	Th2	(81)
Recombinant proteins	ROmpA	66.5 kDa omp	10 μg/ip	MBP	*B. abortus* 544/ 2 × 10^4^/ i.p	IFA	-(3)	2 wks	IgG1↑ IgG2a↑	TNF-α↑ IFN-γ↑ MCP↑ IL-12p70↑ IL-10↑ IL-6↑	Th2	(99)
Recombinant proteins	1- rAspC+rDps rInpB +rNdk 2- MBP	Protein enzyme	20 μg/ip	RB51/ 5× 10^6^/i.p	*B. abortus* 544/ 5× 10^5^/i.p	IFA	Yes	2 wks	IgG2a ↑ IgG1↑	IL-10↑ IL-12p70↑ IFN-γ↑	Th1	(84)
Gene cod (recombinant proteins)	1- rDnaK 2- rTF 3- rOmp31 4- rDnaK+rOmp31 5- rDnaK+rTF	Molecular chaperon, Trigger factor, 31 kDa Omp	30 μg of each/i.p.	Rev1i / 8 × 10^8^/ s.c	*B. melitensis* 16 M / 1 × 10^4^/ i.p	CFA/IFA	Yes	2 wks	IgG2a↑ IgG1↑	IFN-γ↑ IL-12↑ IL-6 ↑ IL-10↓ IL-5↓	Th1	(69)
Recombinant proteins	Rohr	Peroxiredoxin protein	20 μg, i.p	MBP	*B. abortus* 544/ 5 × 10^4^/ i.p	IFA	(-)	2 wks	IgG2a ↑ IgG1↑	IL-10↑ TNF↑ IL-12p70 ↑ IFN-γ↑ IL-6↑ IL-10↑ MCP-1↑	Th1	(67)
Recombinant proteins	1- CobB 2- AsnC 3- Cu-Zn SOD	Pathogenesis-associated proteins	30 μg, i.p	S19 / 1× 10^6^/ i.p	*B. abortus* 544 /2× 10^4^/i.p	CFA/IFA	(-)	2 wks	IgG↑	N.D	N.D	(100)
Recombinant proteins	1- rOmp16+rOmp19+ rOmp28+ rL7/L12 2- pCold-TF	16 kDa Omp 19 kDa Omp 28 kDa Omp Ribosomal protein pCold-trigger factor vector	Protein:100 μg, i.p Vector: 100 μg, i.p	RB51/ 1× 10^6^/ i.p	*B. abortus* 544/ 2× 10^5^/ i.p	IFA	(-)	2 wks	IgG2a ↑ IgG1↑	IFN-γ↑ TNF ↑ IL-6 ↑ MCP-1↑ IL-12p70↑ IL-10↓	Mixed Th1–Th2	(101)
Recombinant proteins	1- BMEI0357 2- BMEI1098 3- BMEI1845 4- BMEII0346 5- BMEII0375 6- BMEII0395	Regulatory proteins of Lrp/AsnC family	30 μg, i.p	S19	*B. melitensis* 16M/ 1× 10^5^/ i.p	CFA/IFA	(-)	2 wks	N.D	N.D	N.D	(76)
Recombinant proteins	1- rL7/L12 2- rBLS 3- L7/L12-B	Ribosomal protein enzyme	-/ s.c, vein of ear	PBS	N.D	Freund's adjuvant	Yes	1 wk	IgG ↑	IFN-γ↑	N.D	(102)
Recombinant proteins (gen cod)	RNdk	Nucleoside diphosphate kinase	20 μg,i.p	MBP	*B. abortus* 544 / 5 × 10^4^/ i.p	IFA	No	2 wks	IgG2a ↑ IgG1↑	IFN-γ↑ TNF↑ MCP1↑ IL-6↑ IL-12p70↑ IL-10↑	Mixed Th1–Th2	(103)
Recombinant proteins (title)	1- Omp19 2- P39 3- Omp19+ P39	19 kDa Omp periplasmic-binding protein	50 μg,i.p	S19 / 1 × 10^4^/	*B. abortus* 544 *B. melitensis* 16M /5 × 10^4^/i.p	CFA/IFA	Yes	2 wks	IgG2a ↑ IgG1↑ IgG2b ↑ IgG3 ↑ IgM ↑	IFN-γ↑ IL-2 ↑ IL-12↑ IL-4↓	Mixed Th1–Th2	(75)
Recombinant proteins (TEXT)	1- rAdk 2- rSecB 3- pcold-TF 4- rAdk+ rSecB	Adenylate kinase Preprotein translocase subunit	50 μg of each /i.p 100 μg/i.p: combined	RB51/ 1 × 10^6^/i.p	*B. abortus* 544/10^5^	IFA	Yes	2 wks	IgG2a ↑ IgG1↑	IL-10↑ IFN-γ ↑ TNF ↑ IL-6↑ MCP-1 ↑ IL-12p70↑	Mixed Th1–Th2	(104)
Recombinant protein (text)	1- rE2o-FA 2- rE2o-Alum	Dihydrolipoamide succinyltransferase	Group1:25 μg/s.c Group2:100 μl/-	S19/1 × 10^4^/ i.p	*B. abortus* 544/ 2 × 10^5^/i.p	CFA/IFA AH	Yes	1 wk	IgG1↑ IgG2a↑ IgG2b ↑	IL-4 ↑ IL-10↑ IFN-γ ↑	Th2	(79)
Recombinant protein (text)	1- rCysK group) 2- rCysK-FA group 3- rCysK-Al	Enzyme	25 μg of each /s.c	S19 / 5 × 10^4^/ i.p	*B. abortus* 544/ 2 × 10^5^/ i.p	CFA/IFA AH	Yes	2 wks	IgG1↑ IgG2a↑	IFN-γ ↑	Th2	(80)
Recombinant protein	1- rRS- 2- rLS-2	Enzyme	100 μg/i.p	Rev1/ 5 × 10^5^	*B. melitensis* 16 M/ 5 × 10^5^/i.p	IFA	Yes	2 wks	IgG ↑	IFN↑ IL-2↑ IL-4 IL-10	Th1	(78)
Epitopic (recombinant proteins)	1- r3E-rIL2 2- rOMP31 3- r3E 4- rIL2 5- chimeric proteins rOMP31-rIL2	Immunogenic epitope of omp31	30 μg,i.p	Rev1/ 1–4× 10^9^ /-	*B. melitensis* M16/ 1 × 10^4^*/i.p*	IFA IL-2	No	2 wks	IgG1↑ IgG2a↑	IFNγ ↑ IL-2↑ IL-4↑	Th1	(89)
Recombinant proteins	1- rOmp25 (40 μg) IP 2- rOmp25 (40 μg) ID	19 kDa Omp	20–30 μg / i.p,id	S19	*B. abortus* 544/10 ^9^/i.p	CFA/IFA	Yes	2 wks	IgG1↑ IgG2a↑	IL-6↑ IL-12↑ TNF↑ IFN-γ↑ IL-4	Th2	(105)
Gene cod (Dna)	SodC+omp19+BLS+ PrpA	Protein enzyme	20 μl / i.n	-	*B. abortus* 544/ 2× 10^4^/	LPS	Yes	-	IgG ↑ IgA↑	IFN-γ↑	-	(86)
Gene cod (recombinant proteins)	1- chimeric protein of Omp19-P39 (rOP)	Truncated 19 kDa Omp Truncated periplasmic-binding protein	50 μl / i.p	S19/ 1× 10^4^/-	*B. melitensis* 16M *B. abortus* 544/ 5 × 10^4^ /i.p	Al CFA/IFA	Yes	2 wks	IgG1↑ IgG2a↑	IFN-γ↑ IL-2 ↑ IL-12p70↑ IL-10 IL-4	Th1	(74)
Recombinant proteins	1- rBLSOmp31-ChM 2- rBLSOmp31-P407-Ch gel 3- rBLSOmp31-IFA	Epitope of 19 kDa Omp Enzyme	500 μg/I.N/CONJ/I.M 0.5 ml/I.N 0.05 ml/CONJ 2 mL/I.M	-	*B. ovis* PA/ 1.09× 10^9^/CONJ, preputial	IFA	Yes	3 wks	IgG↑ IgA↑	IFN-γ↑	-	(106)
Protein	1- OMVs S19 2- OMVs S19 Δ*per*	Protein	15 μg/s.c	S19 /1 × 10^5^/s.c	*B. abortus* 544/ 2 × 10^/^ i.p	-	Yes	-	IgG1↑ IgG2a↑	IL-2↑ IFN-γ↑ TNF ↑ IL-4 ↑ IL-6 ↑ IL-10↑ IL-17A↑	Th2	(107)
DNA vaccine	1- EPLG-Pep 2- APLG-Pep 3- Pep-Ad+ IFA 4- EPLG-NP 5- APLG-NP	MHC-I- andMHC-II-restricted Tcell epitopes formulated by PLG	Group1:50 μg/s.c Group2:100 μg/s.c	DNA vaccine groups: PBS PLG groups: S19/ 5×10^4^/i.p	Group 1: *B.abortus* 544/2× 10/ i.p Group 2: *B. abortus* DB79BRAB4	IFA PLG	Group2: Yes	Group2: 1 wk	-	IFN-γ↑	-	(108)
Recombinant proteins	rL7/L12-Omp25 rL7/L12-Omp25+ rIFN-γ	Fusion protein ribosomal protein + 25 kDa Omp	Protein:30 μg/i.p Cytokine: 10 μg	S19 / 5 × 10^5^/i.p	*B. abortus* 544/ 5 × 10^7^/ i.p	Alum	Yes	-	IgG1↑ IgG2a↑ IgG2b↑ IgG3↑ IgM↑	IFN-γ↑ TNF-α↑ GM-CSF↑ IL-2↑ IL-12↑ IL-5↑ IL-4↓ IL-10↑	Th1	(85)
Recombinant proteins	rOmp28	28 kDa Omp	100 μg/i.p	-	*B. abortus (10^4^)*	IFA	-	2 wks	IgG1↑ IgG2a↑	-	-	(109)
Recombinant proteins	rL7/L12	Ribosomal protein entrapped by PLGA	40 μg/i.p Microparticles: 16 mg	S19/10^5^/i.p	*B. abortus* 544/ 2 × 10^7^/ i.p	Alum MF59	Yes	2 wks	IgG1↑ IgG2a↑	IL-4↑ IFN-γ↑ TNF↑	Mixed Th1–Th2	(110)
Recombinant proteins (gene cod text)	1- rOmp25c	Recombinant unlipidated porin protein	20 μg/i.p	S19/ 5×10^5^/ i.p	*B. abortus* 544/ 5×10^5^/i.p, *B. melitensis* 16M, *B. suis* 1330	CFA/IFA	Yes	2 wks	IgG1↑ IgG2a↑ IgG2b↑ IgG3↑ IgM↑	IFN-γ↑ TNF-α↑ GM-CSF↑ IL-2↑ IL-4↑ IL-5↑	Th2	(83)
Recombinant proteins	r-glk	Enzyme	50 μg/i.m	-	*B. abortus* 544/ 4 × 10^6^/i.p	CFA/IFA	Yes	2 wks	IgG1↑ IgG2a↑ IgG2b↑ IgG3↑ IgM↑	-	Th1	(111)
Gene code	rUrease	Enzyme	20, 30/ i.p., s.c.	S19/10^5^ Rev.1./10^5^	*B. melitensis* 16M, *B. abortus* 544, *B. suis* 1,330/ 2× 10^7^	CFA/IFA	-		IgG1↑ IgG2a↑	IFN-γ↑ IL-12↑ IL-4↓	Mixed Th1–Th2	(112)
Recombinant proteins (title)	1- rSodC+ rRibH+ rNdk+rL7/ L12+rMDH 2- MBP			RB51/1 × 10^6^	*B. abortus* 544/ 5 × 10^4^/i.p	IFA	Yes	1 wk	IgG1↑ IgG2a↑	IFN-γ↑ IL-10↓ IL-12p70 TNF MCP-1 IL-6	-	(113)
Epitope (DNA vaccine)	1- T epitopes 3- B epitopes 3- TB epitopes	T cell and B cell epitopes of OMP31	30 μg/i.n	PBS	Live *B. melitensis* (5 × 10^5^, i.n)	-	-	2 wks	sIgA↑ IgG1↑ IgG2a↑	IFN-γ	Th1	(114)
DNA vaccine	pCIBLSOmp31	1- 31 kDa Omp 2- Enzyme	100 μg/i.m	*B. ovis* PA76250 /1 × 10^9^, *B. canis* M/ 6.3 × 10^8^/	*B. canis* RM6/66/ 5.5 × 10^5^/i.p	IFA AH Montanide Quil A	Yes		IgG1↑ IgG2a↑	IFN-γ↑ IL-4 ↑	Mixed Th1–Th2	(115)
-	1- Fractions B1 2- Fractions B2 3- Fractions B3 4- Fractions B1+B2+B3	Polysaccharide Protein	Group1: 1 μg/i.p Group2: 1 μg/i.m	PBS	*B. suis 145*/ 5 × 10^5^/i.p	-	-	1 wk	IgM ↑ IgG ↑	-	-	(116)
Recombinant proteins (title)	FlgJ FliN	Protein	30 μg/i.p	S19 /1 × 10^5^ /i.p	*B. abortus* 544/ 2× 10^5^/i.p	CFA/IFA	Yes	2 wks	IgG↑	IFN-γ↑	-	(117)
Recombinant fusion	1- rL7/L12 2- TOmp31 3- rL7/L12+ TOmp31	Ribosomal protein Truncated 31 kDa Omp	Fusion protein: 30 μg/s.c Protein:15 μg/s.c	RB51/ 2 × 10^8^/i.p, Rev1 / 2 × 10^8^/i.p	*B. abortus* 544 / 4 × 10^4^/i.p, *B. melitensis* 16M / 2 × 10^4^/i.p	CpG ODN Montanide ISA 50 V	-	3 wks	IgG1↑ IgG2a↑	IL-2↑ IL-10↑ IFN-γ↑	Th1	(118)

*r, recombinant; BP26, recombinant BP26 protein; Omp, outer membrane protein; rMEP, multi-epitope protein; TF, trigger factor; BLS, Brucella lumazine synthase; DnaK, molecular chaperone; Bp26, periplasmic immunogenic protein; p39, sugar-binding 39-kDa protein; L, ribosomal protein; SodC, superoxide dismutase; rPGM, phosphoglucomutase; DapB, dihydrodipicolinate reductase; OMV, Outer membrane vesicle; TOmp2b, truncated outer membrane protein 2b; Th1, T helper1; SOmp2b, short form of Omp2b; BCG, Bacillus Calmette-Guerin; rHSP60, recombinant heat shock protein 60; BLS, Brucella lumazine synthase; FliC, flagellin C; HSDH, hydroxysteroid dehydrogenase; BhuA, outer membrane heme transporter; CpG ODN, CpG oligodeoxynucleotides; pCIOmp31, Omp31 gene cloned in the pCI plasmid; AspC, Aspartate Aminotransferase; Dps, DNA protection during starvation; Ndk, nucleoside diphosphate kinase; DnaK, a cytoplasmic protein; Ohr, hydroperoxide resistance protein; Adk, Adenylate kinase; SecB, a cytoplasmic chaperone; E2o, dihydrolipoamide succinyltransferase; CysK, Cysteine synthase K; FA, Freund's Adjuvant; AL, Alum; RS, riboflavin synthase; LS-2, Loraine synthase; 3E, immunogenic epitope derived from OMP31 antigen; PrpA, proline racemase subunit A; EPLG-Pep, peptides either entrapped in PLG microparticles; Ch, chitosan; APLG-Pep, peptides adsorbed on PLG particles; Pep-Ad, pool of peptides; r-glk, recombinant glucokinase; rRibH, riboflavin synthase subunit beta; rSodC, superoxide dismutase; MDH, malate dehydrogenase protein; OPS, O Polysaccharide; CTB, cholera toxin B subunit; LPS, lipopolysaccharide; CagA, cytotoxin-associated gene A; GI24, 24 amino acids compose the N-terminal α-helical domain; B. melitensis, Brucella melitensis; B. canis, Brucella canis; B. ovis, Brucella ovis; B. suis, Brucella suis; PBS, Phosphate-buffered saline; I.p, Intraperitoneal; S.c, subcutaneous; I.m, intramuscular; CFA/IFA, Complete freund's adjuvant/ Incomplete Freund's adjuvant; TPPPS, Taishan Pinus massoniana pollen polysaccharide; Rev1, Brucella melitensis Rev 1; AH, Antigen-Aluminum Adjuvant; CS-NPs, chitosan nanoparticles; HKBC, heat-killed Brucella canis; B. canis, Brucella canis; MBP, Maltose binding protein; B. abortus, Brucella abortus; MHC, Major histocompatibility complex; Wks, weeks; Ig, immunoglobin; TNF-α, Tumor necrosis factor- α; IFN-γ, Interferon gamma-γ; Th1, T helper type 1; MCP-1, Monocyte chemoattractant protein-1; GM-CSF, Granulocyte-macrophage colony-stimulating factor*.

## DNA Vaccines

DNA-based *Brucella* vaccines are a kind of subunit vaccine which stimulated immune responses following multiple doses (Table 5) (18). These vaccines are safe and efficient brucellosis vaccines due to the stimulation of strong cellular immune responses, expression of several antigens, the existence of CpG motifs, and simple storage conditions (139). DNA-based vaccines contain gene sequences of pathogens, which are essential for intracellular survival of *Brucella* spp. The immunogenicity and efficacy of these virulence genes used in DNA vaccines have been demonstrated in animal studies, including the two-component BvrR/BvrS system (119), Cu-Zn superoxide dismutase (SOD) (126, 140), ribosomal L7/L12 or *Brucella* lumazine synthase (BLS) (139, 141), *B. melitensis* omp31 and omp25 genes (125, 142), antigenic surface protein (BCSP31) gene (120), SP41 (143), and ribosomal protein L9 (rL9) (122). According to the studies that have been done, DNA vaccines may have the ability to resolve the disadvantages of other brucellosis vaccines (119, 120, 144). In most studies, animals vaccinated with different types of DNA vaccines have shown full protection against virulent strains (e.g., *B. abortus S19, B. abortus 2308, B. melitensis 16M*, and *B. melitensis Rev1*) (120, 143).

**Table 5:** DNA and nanoparticle- based vaccines.

**Name of vaccine**	**Type of vaccine**	**Structure of vaccine**	**Advantage**	**Disadvantage**	**References**
B. abortus S19	DNA-based	pCDNA-BvrR (plasmid pCDNA-BvrR)	• Conferring a significant level of protection in animals due to inducting a specific Th1 response (antibody), increased IFN-γ expression level compared with IL-4, and a strong T cell-proliferative response • BvrR is a promising candidate for studies on DNA vaccines against brucellosis in the future.	• Lower antibody titers in pCDNA-BvrR vaccine group compared with other constructed DNA vaccines against *Brucella*	(119)
B. abortus	DNA-based	DNA encoding antigenic surface protein (BCSP31)	• Exhibiting a protective efficacy in rabbit models due to inducting appropriate cellular immune responses	NR	(120)
*B. abortus* 2308	DNA-based	DNA encoding the BAB1 0263 (pVF263) and BAB1 0278 (pVF278)	• In animals, both vaccines elicit a T-cell response (cellular immunity) and a dominant IgG2a response (humoral responses). • Only pVF263 induces significant levels of INF-γ. • None of them induce IL-10 and IL-4. • A significant protection is induced by BAB1 0278 antigen.	• Inability of pVF278 to stimulate significantly the production of cytokines, particularly IFN-α • Inability of pVF263 plasmid to confer a significant level of protection compared to pVF278 DNA	(121)
*B. melitensis* 16M	DNA-based	DNA-SP41 vaccine	• Inducing SP41-specific serum IgG antibodies • Inducing a T-cell proliferative response and IFN-γ production (Th1) but not IL-5	• Vaccination with Rev1 induces better and sufficient protection levels than SP41 DNA vaccine against *B. melitensis* 16M in mice	(120)
*B. abortus*	DNA-based	Plasmid DNA vaccine encoding ribosomal protein L9	• Increasing IgG antibody responses (both IgG1 and IgG2a isotypes) • Inducing the secretion of Th1-type cytokines: IFN-γ by CD4+ and CD8+ T cells as well as TNF-α and IL-2 but not IL-4 • Following the EP and prime/boost strategy induces protection against *B. abortus* infection compared to S19 vaccine.	NR	(122)
Wild-type *B. abortus*	DNA-based	Recombinant GntR plasmid (pVGntR)	• Inducing more significant protection compared to conventional RB51 vaccine by increasing IgG as well as IFN-γ and IL-4 (Th1- and Th2- immune responses)	NR	(123)
*B. abortus* 2308	DNA-based	Recombinant plasmid based on BAB1-0267 and BAB1-270 ORFs (encodes protein with SH3 domain and Zn dependent metalloproteinase)	• BAB1_0267 ORF: significantly increases the production of IgG1 and IFN-γ as well as Th1-type immune responses. • BAB1_0270 ORF: is considered as an effective candidate due to significantly increasing the production of IgG2a, IFN-γ, and TNF-α as well as Th1-type immune responses.	• BAB1_0267 does not provide significant levels of protection	(124)
*B. melitensis* Rev1	DNA-based	pcDNA3.1-Omp25-31	• Increasing the levels of IgG and IFN-γ as well as inducing a T-cell-proliferative response • Eliciting strong and protective humoral and cellular immunity	NR	(125)
*B. abortus* 2308	DNA-based	Multi-epitope DNA vaccine encoding epitopes from Cu-Zn SOD	• Eliciting IgG, IFN-γ, and Th1 responses but no IL-4 • Inducing humoral and cellular immune responses in animals	• The production of IL-4 as an indicator of Th2 activation is not detected	(126)
*B. abortus* 2308	DNA-based	Multivalent DNA vaccines by fusion of BAB1 0273 and/or BAB1 0278 of ORF from GI-3 and *B. abortus* 2308 *sodC*	• Inducing both humoral and cellular immune responses • Inducing a significant increase in the production of IgM, IgG, IgG2a, and IFN-γ, as well as Th1-type immune responses	• Inducing low protection levels in mice challenged with *B. abortus* 2308	(126)
*B. abortus* 2308	DNA-based	DNA vaccine containing ORF of GI-3 with ABC-type transporter (pV278a)	• Conferring protection in animals due to increasing the secretion of dominant IgG2a and INF-γ but not IL-4	NR	(127)
*B. ovis*	Nanoparticle- based	Mannosylated nanoparticles (MAN-NP-HS)	• Significantly conferring a higher protection level than Rev1 due to eliciting a more intense mucosal IgA response and elevating IL-2, IL-4, and IFN-γ levels. • MAN-NP-HS is distributed from palpebral area to the nasal region and the GI tract. • As a safe and suitable adjuvant for conjunctival vaccination	NR	(128)
*B. abortus*	Nanoparticle- based	• Malate dehydrogenase (rMdh), rOmp 10 and 19 loaded in mucoadhesive • CNs	• Inducing an increase in IgG especially IgG1, IFN-γ, and IL-4 (Th1-Th2 response) levels • Significantly increasing anti-Mdh IgA in nasal and fecal excretions, and anti-Omps IgA in sera, nasal, and genital secretions and fecal excretions • Increasing anti-Mdh IgA antibody level but not anti-Omps IgA • Inducting antigen-specific IgA, Th2-polarized immune responses, and highly specific IgG	NR	(129)
*B. melitensis* 16 M and *B. abortus* 544	Nanoparticle- based	Chimeric antigen TF/Bp26/Omp31 (TBO) loaded glycine nanoparticles	• Inducing high levels of IgG and IgA in immunized mice sera and mouth • Inducing both cellular and humoral immune responses • i.p administration could generate a better immune response in comparison with oral and nasal administration as well as antigens-Freund adjuvant administration.	• Oral administration fails to induce the highest level of protection against *B. melitensis* 16 M and *B. abortus* 544 in comparison with i.p injection of nanovaccine	(130)
*B. abortus* 544	Nanoparticle- based	OPS and LPS antigens conjugated with PLGA nanoparticles (LPS-PLGA and OPS-PLGA)	• Both improve the immunization process in animals and humans against brucellosis due to inducing IgM and IgG secretion and more protection than pure antigens (OPS and LPS). • LPS-PLGA conjugate vaccine induces more immunogenicity compared to OPS-PLGA nanovaccines.	NR	(131)
*B. abortus*	Nanoparticle- based	Malate dehydrogenase (Mdh), loaded in mucoadhesive CNs (CNs-Mdh)	• Inducing higher IL-6 production than unloaded antigens and TF loaded CNs (CNs-TF) • Significantly increasing IL-4 and IgG-secreting cells after 4W • Increasing Mdh-specific IgG levels after 6W (IgG1 and IgG2a but with the predominance of IgG1 response) • Inducing a significant increase in Mdh-specific IgA and total IgA in secretions and sera of immunized group • Intranasal immunization effectively induces antigen-specific mucosal immune responses through the elicitation of Th2-related immune responses.	NR	(132)
*B. melitensis* and *B. abortus*	Nanoparticle- based	Trimethyl chitosan nanoparticles of Urease (TMC/Urease)	• Eliciting low titers of specific IgG following i.p injection of urease alone and oral administration of both TMC/Urease and urease alone • Inducing high levels of specific IgG following i.p administration of TMC/Urease • Eliciting a Th1-Th2 immune response following i.p administration of urease alone and TMC/Urease • Eliciting a Th1-Th17 immune response following oral administration of urease alone and TMC/Urease • Inducing a cell proliferative response in spleen cells of i.p vaccinated mice with TMC/Urease nanoparticles • i.p vaccination with TMC/Urease nanoparticles results in a high degree of protection.	• Failing to induce the highest level of protection against virulent strains of *Brucella* spp. due to not eliciting a detectable specific IgA immune response • Inducing a lower degree of protection than i.p. immunization	(133)
*B. melitensis* 16 M and *B. abortus* 544	Nanoparticle- based	Mannosylated Chitosan Nanoparticles (MCN) loaded with *FliC* protein	• Inducing a significant increase in specific IgG (higher IgG2a titers), IgM, and IgA levels; high levels of IFN-γ and IL-2; but low levels of IL-10 following FliC and FliC-MCN challenges • Conferring a significant level of protection due to humoral and cellular responses of Th1-dominant type as well as cross protection against *B. melitensis* and *B. abortus* infections	• Conferring less protection than live attenuated *B. melitensis* Rev1 and *B. abortus* RB51 vaccines against *B. melitensis* 16 M and *B. abortus* 544	(134)
*B. melitensis* 16 M and *B. abortus* 544	Nanoparticle- based	Calcium phosphate nanoparticles (CaPNs)	• Eliciting increased ratio of specific IgG2a to specific IgG1, high levels of IFN-γ and IL-12 (cellular and humoral immune responses), and low levels of IL-10 • Conferring protection against *B. melitensis* 16M and *B. abortus* 544 • All antigens used in the vaccine formulations are conserved between *B. melitensis* 16M and *B. abortus* 544; therefore, cross protection could be obtained by a single vaccine.	NR	(135)
*B. melitensis* 16M	Nanoparticle- based	Omp31-loaded N-trimethyl chitosan nanoparticles (TMC/Omp31)	• Oral immunization induces a Th1–Th17 immune response but lower antibody titer. • i.p immunization by Omp31-IFA and TMC/Omp31 NPs induces Th1 and Th1–Th2 immune responses and high IgG titer (IFN-γ and IL-12). • Only in i.p administration route of TMC/Omp31, high IL-4 production vaccination with Omp31 stimulates a vigorous cell proliferative response which could further be increased with oral immunization with TMC/Omp31 NPs. • Conferring a significant level of protection in the orally administered group in comparison with the i.p immunized mice due to Th17 response	NR	(136)
*B. abortus* 544	Nanoparticle- based	L7/L12 entrapping PLGA nanoparticles	• Inducing high IgG antibody titers (predominant IgG1; however, IgG1/2a ratio shows a mixed profile of Th1/Th2 responses.) • Inducting high levels of Th1 cytokines, especially IFN-γ • Potently inducing an inflammatory cellular response • Inducing a significant reduction in CFU of splenic bacteria in the vaccinated mice against *B. abortus* 544 • Inducing both humoral and cellular responses	NR	(137)
*B. melitensis* 16M	Nanoparticle- based	Nanovaccines against based on PLGA nanoparticles and oligopolysaccharide antigen	• Inducing a significant increase in IgG and IgM titers and efficient opsonophagocytosis of *Brucella* in the sera of immunized animals • Conferring a high level of protection • Could be considered as a candidate for immunization of animals and humans against the diseases caused by *B. melitensis* and needs further investigations	NR	(138)

*Introduced DNA vaccines*.

*NR, not reported; IFN, interferon gamma γ; IL, interleukin; ORFs, open reading frames; GI-3, genomic island 3; Th1, T-helper 1; IgG, immunoglobulin G; SOD, superoxide dismutase; GI3, genomic island; rOmp, outer membrane proteins; CNs, chitosan nanoparticles; W, weeks; i.p, intraperitoneal*.

Intramuscular (i.m.) administration of DNA-based vaccine has been shown to induce a protective immune response as similar as Rev1 in different animal model studies (125, 143, 145, 146). Jain et al. demonstrated that the electroporation (EP) approach induced further protective responses than the i.m. route (122). A combination of several suitable antigens, such as L7/L12, BCSP31, SOD, P39, and omp16, could be used to make a “divalent or poly-antigenic DNA vaccine,” which has been reported to be effective due to more antigenic components, induction of a wide range of humoral and cellular immune responses, and simulation of the most similar status to *Brucella* infection (78, 126, 143, 147–149).

Same as subunit vaccines, DNA-based brucellosis vaccines can stimulate both humoral and cellular immune system arms, TCD4 and TCD8 helper cells, as well as a significant increase in IFNγ, TNF-α, and IL-12 levels (122), which IFNγ exerts a protective effect by boosting macrophage activity (150).

However, several publications have shown no changes in the expression of IL-4, IL-10, and IFN-γ following DNA vaccine administration which may be related to the suppressive function of Treg in preventing IFN-γ development (151–154). DNA-based vaccines do not provide significant levels of protection compared to live-attenuated vaccines. This is consistent with the finding of Kurar et al., Leclerq et al., and Schurig et al. studies which indicated that a lower immune response and no protective response was observed following the administration of DNA based vaccine against *Brucella* challenge (7, 155, 156). It may happen due to the inability of the vaccine to express specific antigens such as GroEL-Hsp antigen in PcDNA3-DNA vaccine (7). The need for repeated booster doses administration in response to the rapid silencing of genes, is the main reason for the loss of long-term protective response which could be improved using an adjuvant. This result is in line with the finding of a study by Velikovsky et al., demonstrating that following repeated vaccination with PcDNA3 containing the BLS gene, a protective response was induced in mice in addition to the production of IgG2a (157). Therefore, despite the expression of protective antigens, DNA-based vaccines may unable to express antigens in high amounts, and today efforts are made to delay gene silencing for a longer time.

## Nanoparticle Based Vaccines

Nanoparticles (NPs) containing *Brucella* vaccine induce antibody responses (IgM, mucosal IgA, and IgG) (129, 130), increase IFN-γ, IL-12, IL-4, and IL-6 levels, and decrease IL-10 levels (134, 135) in animal model studies (Table 5). Most studies have reported increased IgG1 level linked to the Th2 response, compared to IgG2 level (129, 134, 135, 137) which is linked to the Th1 response (135). Nanoparticle-based vaccines cannot be used to vaccinate humans against brucellosis due to the risk of disease (138), however, oral vaccines show more benefits in an animal model study (133). In addition to a Th1-Th2 response (129, 130, 132), oral administration of NP-based vaccines induces a Th1-Th17 response which is stronger and suitable for controlling brucellosis. Despite many advantages of oral vaccines over intraperitoneal (i.p) vaccines, including ease of preparation, painless administration, and a stronger Th1-Th17 response induction (133, 158), they are less effective in inducing antibody responses, especially IgA. Relative toxicity, limitation in both antigen loading and vaccine production as well as weak stimulation of the immune system are the most disadvantages of nanoparticle-based vaccines (159, 160). According to animal model studies, a decrease in the number of CFUs of splenic bacteria is observed following NP-based vaccines administration, indicating that these vaccine have the potential to induce protection against brucellosis. The immune response induced by NPs depends on their uptake by antigen-presenting cells (APCs) and their particle size and charge (137). A powerful protective response needs a combination of Th1 and IgA responses (135).

Mannosylation of nanoparticles in the MAN-NP-HS vaccine candidate aids nanoparticles to reach directly mannose receptors that are abundantly expressed on the surface of immune cells and are important in antigen uptake. Following administration of the MAN-NP-HS vaccine, a mixture of mucosal IgA antibodies and Th1-Th2 cytokines including IL-12, IL-4, and IFN-γ is produced, of which IFN-γ plays a critical role in inducing cellular immunity. According to these findings, MAN-NP-HS provides even more protection than Rev1 due to the induction of more specific IgA (131). This vaccine, which is administered through the eye (palpebral), shows no side effects or inflammation. Moreover, the release of the MAN-NP-HS vaccine from the palpebral to the nasal mucosa and GI tract leads to greater protection (36). Another candidate is a combination of LPS and OPS antigens with PLGA nanoparticles, which has the potential to induce strong protection in animals and humans by producing IgM and IgG antibodies. These antigens alone are not effective in inducing immunity, but when combined with nanoparticles, they produce more antibodies (156). Most effective nanovaccine candidates induce a significant reduction in bacterial load in splenocytes, Th1 response, and antibody response, especially mucosal IgA. Choosing an antigen that is protected between two pathogenic strains is critical because it contributes to the induction of cross-protection against both strains following vaccination (135).

## Other Vaccines

*Brucella* dual vaccine is a new approach to the development of a *Brucella*-based vaccine platform of immunogenic antigens, oriented to simultaneously control the transmission of two important bacterial pathogens from cattle to humans. In a study by Abedi et al., the use of a total TN-OMP (outer membrane vesicles of *Brucella*) conjugated with rCagA (recombinant protein of *Helicobacter pylori*) was evaluated, and the results revealed that rCagA as an adjuvant increased the immune response against TN-OMP. Thus, this combination vaccine was effective in inducing simultaneously serum bactericidal and splenic activities of *B. abortus* and *H. pylori* in BALB/c mouse model (161). Similarly, Bahador et al. showed that subcutaneous immunization of mice by conjugated rCagA with *Brucella* LPS (rCagA+ LPS) induced protective effects. Iannino et al. designed the Bab-pgm strain (genetically engineered live *B. abortus* vaccine) as a heterologous carrier for the recombinant chimeric antigen to deliver Shiga toxin-producing *E. coli* (STEC) in a mouse model, which resulted in the induction of a protective immune response against two very different pathogens (162). Another approach to vaccine development is the use of a modified *Brucella* immunodominant antigen instead of deleting *Brucella* antigens or epitopes or introducing a foreign antigen, which could induce differential antibodies against *B. ovis* (163). Another approach to vaccine production is polysaccharide conjugate vaccines which are produced *via* the covalent glycan-protein conjugation of bacterial surface; they have been proven to be cost-effective tools to prevent dramatic infectious diseases. It has been demonstrated that OPS of *B. abortus* could be expressed in *Yersinia enterocolitica O:9* and displayed on CTB *via* glycosylation, eliciting an antigen-specific immune response and a significant protection level against brucellosis (164). Antigen-delivery systems, such as attenuated viruses or bacteria, are essential for presenting *B. abortus* immunogenic antigens to the immune system cells. Recently, Lin et al. designed an adenoviral vector expressing both p39 and lumazine synthase proteins of *B. abortus*, which elicited significant humoral and cellular immune responses, although pre-existing immunity against adenovirus could prevent a vaccine from working (165). There are several studies using liposomes as *Brucella* antigen-delivery systems. Liposomes are not only widely used as a carrier to improve vaccine efficacy and efficiency in the transport of antigens to appropriate sites but also possess adjuvant properties against bacteria (166). Goel et al. showed that liposome-encapsulated recombinant Omp25 induced a protective immune response comparable to that induced by S19 in a mouse model (167). In another study, subcutaneous co-administration of *Brucella* Cu-Zn SOD recombinant protein with recombinant IL-18, encapsulated in *E. coli* lipid liposome (escheriosome), demonstrated a stronger IgG2a-type antibody response in immunized mice compared with free BaSOD DNA. Another approach to vaccine development against *Brucella* infection includes lysed *B*. *abortus* (168, 169) or whole organism of *Brucella* spp. without cytoplasmic contents. The bacterial-ghost (BG) technology is the use of biologically killed Gram-negative bacterial cells produced *via* controlled expression of the cloned lysis gene *E* of X174 bacteriophage. BGs are potential envelope structures lacking cytoplasmic contents, which act as a delivery system and an efficient adjuvant for DNA- and protein-based vaccines. In the case of *Brucella*, it has been reported that *Brucella S2* ghosts effectively elicit a pathogen-specific antibody response, enhancing IgG antibody and T cell responses in mice compared to inactivated bacteria (170). Kwon et al. used a fragment of PMPA-36 (porcine myeloid antimicrobial peptide 36), named GI24, for *B. abortus* lysis and produced *B. abortus* ghosts, termed as *B. abortus* lysed cells (171). Due to the lack of genetic materials in BG vaccines, the horizontal transfer of antibiotic resistance genes or pathogenic islands to the resident gut flora does not occur.

## Future Trends of Brucellosis Vaccines

There are many efforts for the development of new vaccines, safer and more effective based on new technologies such as the engineered live-attenuated vaccines based on deletions in virulence genes, and viral or bacterial vector-based *Brucella* vaccines, subunit vaccines, DNA vaccines, Nanoparticle-based vaccines. The majority of these vaccines designed with regard to new technologies showed the enhanced immune responses and protective immunity against brucellosis in mice, livestock, and guinea pig models. For example, Bugybayeva et al. (52) indicated that the tetravalent vaccine formulation Flu-NS1-80-Omp16+Flu-NS1-80-L7/L12+Flu-NS1-80-Omp19+Flu-NS1-80-SOD protected guinea pigs from B. melitensis 16M infection at a significant level (*P* < 0.05). Thus, this vaccine can be chosen as a potential vaccine candidate for further development of an effective human vaccine against brucellosis.

The subunit vaccines are promising vaccine candidates due to their safety profile, well-defined non-infectious nature, inability to revert to a virulent strain, non-viability unlike attenuated vaccines, and capability of manipulation to maximize desirable activities. However, they indicated some disadvantages such as the poor antigenicity, instability, and short half-life of recombinant subunit antigens. Hence, the use of adjuvants, immunomodulators, and antigen delivery systems, or is necessary to enhance immune responses. For these reasons, already despite many efforts, no successful subunit vaccine has been developed for brucellosis livestock (172). On the other hand, DNA vaccines are one of the methods employed for developing a safe and efficient brucellosis vaccine due to stimulation of cellular immune responses and expression of several antigens; however, they do not induce significant levels of protection due to the lack of a long-term protective response (157).

Out of vaccines with new technologies, the engineered live-attenuated vaccines based on deletions in virulence genes have accounted as the best approach for developing new vaccines with minimal residual virulence, due to the induction of high safety levels compared to classical live-attenuated vaccines. A variety of these vaccine types are under development based on different deletions in *B. abortus* or *B. melitensis* virulence genes, which eventually result in significant attenuation and increased production of T cells, pro-inflammatory cytokines, and antibodies (19). Hence, they can be considered a promising vaccine candidate for human use.

## Conclusion

To date, no vaccine licensed against human brucellosis is available. Hence, the control of human brucellosis has relied heavily on the control of animal brucellosis by vaccination. Live-attenuated vaccines such as *B. abortus* strains S19 and *B. melitensis* strain Rev1 as the two most common anti-brucellosis vaccines have been widely used in the world for the immunization of animals. However, they had some drawbacks, such as the induction of abortion in pregnant animals, the virulence for humans, the production of anti-*Brucella* antibodies interfering with serodiagnosis, and the antibiotic resistance against brucellosis treatment. Two factors should be considered in designing an effective brucellosis vaccine: the route of vaccine administration and the design of the vaccine to induce cell-mediated immunity which is the most important component of the immune system in inducing defense. It appears that of the brucellosis vaccines, the live attenuated vaccines that some of their genes have been deleted are more effective. They can increase the production of T cells, pro-inflammatory cytokines, and antibodies. Therefore, they can be considered a promising brucellosis vaccines.

## Author Contributions

MH, SD, RG, MM, NB, AD, TN, and MT contributed in revising and final approval of the version to be published. All authors agreed and confirmed the manuscript for publication.

## Conflict of Interest

The authors declare that the research was conducted in the absence of any commercial or financial relationships that could be construed as a potential conflict of interest.

## Publisher's Note

All claims expressed in this article are solely those of the authors and do not necessarily represent those of their affiliated organizations, or those of the publisher, the editors and the reviewers. Any product that may be evaluated in this article, or claim that may be made by its manufacturer, is not guaranteed or endorsed by the publisher.
